# Neonatal presumed septic pulmonary embolism associated with a peripherally inserted central catheter: a case report

**DOI:** 10.3389/fped.2026.1945019

**Published:** 2026-09-23

**Authors:** Chun-fang Gao, Dan Sun, Ling-Kong Zeng, Xu-wei Tao

**Affiliations:** Wuhan Children’s Hospital (Wuhan Maternal and Child Healthcare Hospital), Tongji Medical College, Huazhong University of Science & Technology, Wuhan, China

**Keywords:** neonate, next-generation sequencing, peripherally inserted central catheter, pulmonary abscess, septic pulmonary embolism

## Abstract

Septic pulmonary embolism (SPE) is a rare complication of peripherally inserted central catheters (PICC), with few neonatal reports. We present a premature infant who developed recurrent apnea and hypoxemia during PICC indwelling. Imaging revealed bilateral thick-walled cavitary lesions. Although two consecutive conventional peripheral blood cultures remained negative, MSSA was identified from PICC tip culture, deep sputum culture, and NGS analysis of peripheral blood, PICC intraluminal blood, and deep sputum samples. The infant recovered after 39 days of antimicrobial therapy, including five weeks of targeted linezolid. This case suggests that PICC surface biofilms were the most probable source of septic emboli causing occult SPE, underscoring the importance of considering catheter-related pulmonary embolism in neonates with unexplained respiratory deterioration.

## 1 Introduction

Septic pulmonary embolism (SPE) (1) is a critical condition resulting from infected thrombi lodging in the pulmonary arteries, causing pulmonary infarction, vasculitis, and tissue necrosis. In adults, SPE is commonly associated with intravenous drug abuse, central venous catheters, infective endocarditis, and deep tissue infections; while in children, the incidence is lower and typically secondary to underlying diseases or medical interventions (2). *S. aureus* and *Candida* species are the predominant pathogens, with strong invasiveness leading to vascular damage and distant dissemination (3, 4). Clinically, SPE often presents with non-specific symptoms such as fever and cough and is frequently mistaken for common pneumonia. However, delayed intervention can rapidly progress to life-threatening complications, including necrotizing pneumonia and septic shock, and is associated with high mortality (5). Therefore, early recognition and precise intervention are essential. Central venous catheters are widely used in critically ill neonates, and common complications such as catheter obstruction, local infection, and catheter-related bloodstream infection have received considerable clinical attention (6). However, catheter-associated SPE is rarely reported owing to its low incidence. Here we present a case of presumed SPE associated with PICC-related methicillin-susceptible *S. aureus* infection, which progressed to bilateral pulmonary abscesses in an extremely low birth weight infant, aiming to provide a clinical reference for the early identification and precise management of this rare complication.

## 2 Case report

A female infant delivered by cesarean section at 27 weeks and 6 days of gestation had a birth weight of 990 g (10th percentile) and Apgar scores of 5 at 1 min and 7 at 5 min. She was admitted to the neonatal intensive care unit (NICU) immediately after birth due to respiratory distress, and received nasal Continuous Positive Airway Pressure (nCPAP) and parenteral nutrition. Enteral feeding was initiated on day 1 but was poorly tolerated, presenting with vomiting and abdominal distension. An umbilical venous catheter (UVC) was placed for the first 7 days. On postnatal day 8, the UVC was removed and replaced with a peripherally inserted central catheter (PICC). Under color Doppler ultrasound guidance, the PICC was inserted via the superficial temporal and external jugular veins into the subclavian vein and subsequently into the superior vena cava, without complications (Figure 1A). After admission, the infant's respiratory status gradually improved, and oxygen delivery was stepped down from nCPAP to heated humidified nasal cannula (HHNC, FiO_2_ 25%, flow 4 L/min), with stable oxygen saturation. On day 14 after PICC insertion (postnatal day 21), the infant developed 3-5 apneic episodes per hour with hypoxemia (SpO_2_ < 85%), requiring an increase in FiO_2_ to 40%–50%, and escalation of respiratory support from HHNC to nCPAP (PEEP 6–8 cmH_2_O), accompanied by lethargy, poor responsiveness, and reduced limb tone.

**Figure 1:**
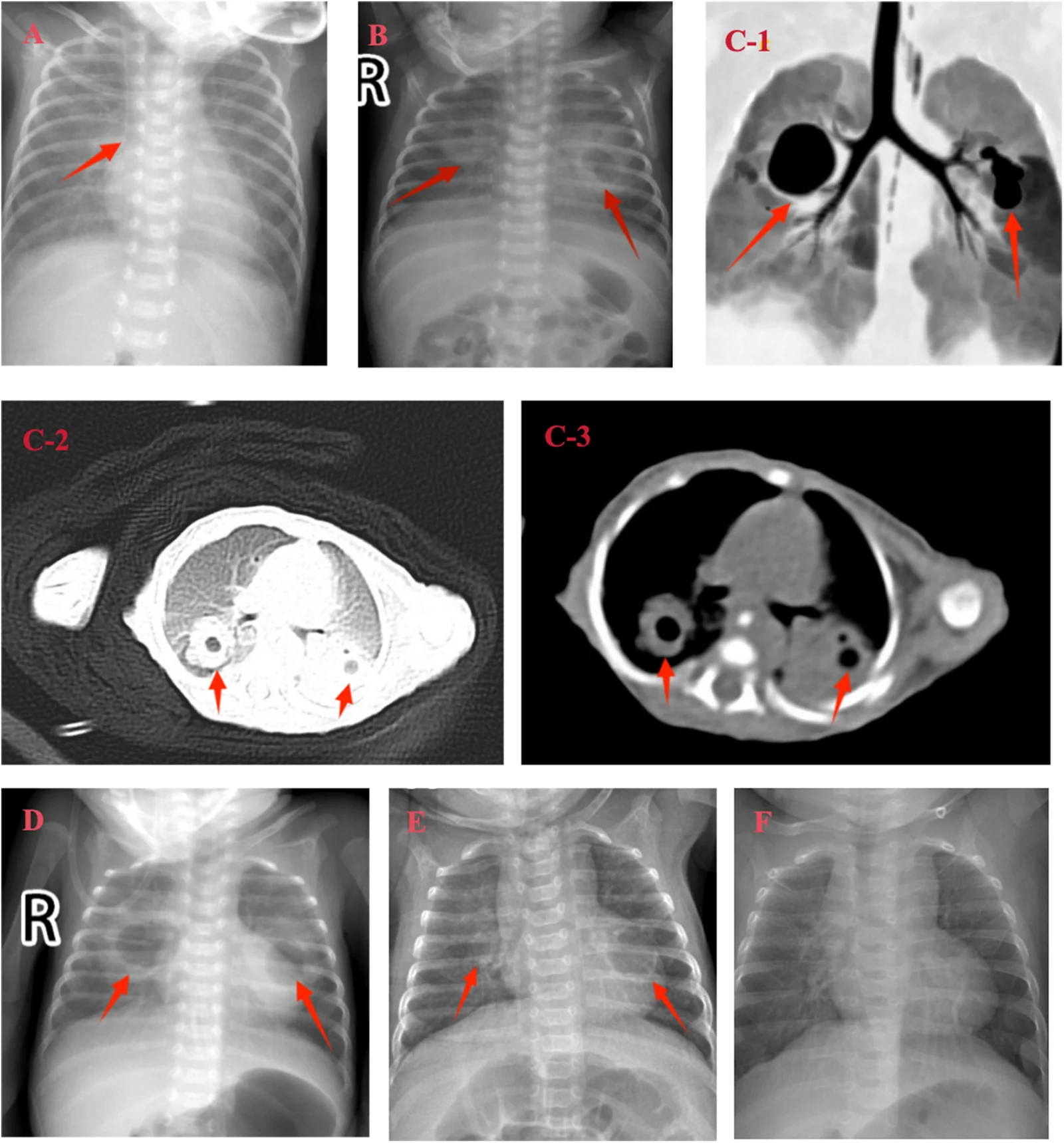
Imaging findings of the infant. **(A)** Chest x-ray (PICC day 8, postnatal day 16): Proper PICC placement. **(B)** Chest x-ray (PICC day 14, postnatal day 21, onset of clinical symptoms): Patchy consolidations in both lungs with thick-walled cavities. **(C)** C1-2-3: Contrast-enhanced chest CT(1 day after clinical onset, postnatal day 22): Bilateral pneumonia with lung abscess formation (C-1: coronal view; C-2 and C-3: axial views at different levels). **(D)** Chest x-ray (treated with 4 days vancomycin and 1 day linezolid, postnatal day26): Slight progression of the right-sided lesion. **(E)** Chest x-ray (1week after drug discontinuation, postnatal day 67): Significant absorption of lung lesions and reduction in cavity size. **(F)** Chest x-ray (2 months after treatment discontinuation): No obvious abnormalities.

Chest x-ray revealed bilateral diffuse patchy consolidations with thick-walled cavities and surrounding exudative changes, suggestive of bilateral pulmonary abscesses. Contrast-enhanced chest CT showed: ① multiple bilateral thick-walled cavitary lesions, with the largest measuring approximately 14.7 mm × 10.2 mm in the right lung and 20.3 mm × 12.1 mm in the left lung, with slightly irregular inner walls and patchy peripheral exudative opacities (Figure 1); ② a well-defined, round nodule (approximately 3 mm) in the periphery of the right lung without significant contrast enhancement; ③ dense consolidation in both lower lobes with thickened and distorted pulmonary markings; and ④ localized emphysema in the left lower lobe. Laboratory parameters showed a peripheral leukocyte count was 21.11 × 10^9^/L (reference range: 5.0–20.0 × 10^9^/L), with neutrophils count was 15.54 × 10^9^/L (reference range: 2.0–10.0 × 10^9^/L); platelet count was 299 × 10^9^/L (reference: 242–378 × 10^9^/L); C-reactive protein (CRP) was 4.3 mg/L (reference: 0–3 mg/L). Serum 1,3-*β* D-glucan (G-test), galactomannan (GM-test), T-SPOT, TB assay, and nucleic acid testing for respiratory syncytial virus, adenovirus, influenza virus, and parainfluenza virus were all negative. Ultrasound of the heart and the vessels showed no obvious vegetation or thrombus formation. To exclude central nervous system involvement, a lumbar puncture was performed on postnatal day 21 prior to antibiotic administration. CSF analysis showed normal cell count and differential, protein, chloride, glucose, and negative bacterial culture, effectively ruling out meningitis.

Microbiological Findings including: ① Peripheral blood culture: 2 consecutive samples were negative; ② PICC tip and deep sputum cultures: methicillin-susceptible *S. aureus* (MSSA) was isolated; ③ Next-generation sequencing (NGS): using the Illumina NovaSeq 6,000 platform. Samples (peripheral blood, PICC intraluminal blood, and deep sputum) were collected at the onset of clinical deterioration (PICC day 14, postnatal day 21), before the initiation of antibiotic therapy. Deep sputum specimens were obtained by passing a sterile suction catheter through the nasal cavity into the lower airway under aseptic conditions, with suction applied only after the catheter had bypassed the oropharynx to minimize upper-airway contamination. The procedure was performed by experienced neonatal nurse. Each sequencing run included sterile water as a negative control, and no microbial reads were detected in these controls. The validated detection threshold for a positive call was defined as ≥5 specific reads mapping to a given pathogen at the genus or species level, with a relative abundance of ≥0.5% after stringent host-background subtraction (removal of human reads). This threshold was established and validated by the performing laboratory through serial dilution experiments using reference strains (including *S. aureus*, *E. coli*, and *C. albicans*) spiked into negative clinical matrices at varying concentrations to determine the limit of detection, following the general principles outlined in the expert consensus on the clinical application of metagenomic next-generation sequencing for pathogen detection (7). The ≥5 read count threshold was chosen to balance sensitivity and specificity by minimizing false-positive calls from low-level background noise or reagent contamination, while the ≥0.5% relative abundance criterion helped distinguish true pathogens from commensal or environmental organisms (8). Clinical significance was assigned when the same pathogen was concordantly detected in at least two distinct sample types in the context of compatible clinical and imaging findings. After rigorous host-background subtraction and quality filtering, *S. aureus* was detected in all three specimen types with concordant signals (specific read counts: 156 in peripheral blood, 243 in PICC intraluminal blood, and 892 in deep sputum; relative abundance >5% in each specimen). Antimicrobial susceptibility testing showed that the isolate was mecA gene-negative and susceptible to cefoxitin, linezolid, and vancomycin, confirming *MSSA* as the causative pathogen.

The PICC was removed immediately after microbiological sampling on postnatal day 21, before the initiation of antimicrobial therapy. The infant initially received vancomycin for 4 days, with the dose escalated from 15 mg/kg to 20 mg/kg every 12 h. However, trough concentrations remained subtherapeutic (3.06 μg/mL and 3.78 μg/mL, respectively), and the infant showed no clinical improvement; pulmonary lesions progressed radiographically. After MSSA was confirmed, the persistently subtherapeutic vancomycin trough concentrations despite dose escalation, coupled with clinical deterioration, prompted a multidisciplinary reassessment. Given this infant's extremely low birth weight and gestational immaturity, the rapid onset of severe bilateral pulmonary abscesses, and our institutional experience, the regimen was switched to linezolid as a pragmatic alternative therapy, considering its reliable lung penetration, favorable bioavailability, and after multidisciplinary discussion (involving neonatology, pediatric pulmonology, and clinical pharmacy), our team reached a consensus to switch to linezolid (10 mg/kg every 8 h) as a pragmatic, individualized alternative rather than standard first-line therapy. One week later (postnatal day 32), apneic episodes resolved, breathing stabilized, and oxygenation remained stable on nCPAP (FiO_2_ 30%, PEEP 5 cmH_2_O). At 2 weeks of treatment (postnatal day 39), repeat imaging showed marked reduction in the size of bilateral pulmonary abscesses, thinning of cavity walls, and resolution of surrounding exudates. On HHNC (FiO_2_ 25%, flow 3 L/min), the infant had stable respiration with normal oxygen saturation. The infant subsequently completed a 20-day oral course of linezolid. The transition to oral therapy was based on three considerations: (1) the infant showed significant clinical improvement with resolution of apneic episodes and stabilization of respiratory status, accompanied by radiographic evidence of marked reduction in pulmonary abscess size and normalization of infection markers; (2) the infant had achieved full enteral feeding with good tolerance, stable weight gain, and no vomiting or abdominal distension, confirming that gastrointestinal absorption was clinically adequate; and (3) switching to the oral route minimized additional invasive procedures and reduced the risk of complications associated with prolonged intravenous access in this extremely low birth weight infant. During the 35-day linezolid course, the infant was closely monitored for potential adverse effects. Serial complete blood counts, liver function tests, and renal function parameters were obtained weekly. The infant tolerated the prolonged course well without clinical evidence of adverse drug reactions.

During the disease course, serum CRP, leucocyte count, and neutrophil percentage increased, whereas platelet count remained normal (Figure 2A). After 15 days of intravenous therapy (vancomycin 4 days and linezolid 11 days, postnatal day 35), serial infection markers normalized. Postnatal day 56, the infant was weaned from supplemental oxygen and maintained regular spontaneous breathing with normal oxygen saturation. Total antimicrobial therapy was 39 days: vancomycin IV for 4 days, followed by linezolid IV for 15 days and linezolid oral for 20 days. The infant was then observed for one week without clinical abnormalities and discharged. At 2-month follow-up, the infant had normal growth and development, complete resolution of pulmonary lesions, and no sequelae. Imaging findings are presented in Figure 1, and the clinical timeline is shown in Figure 2B.

**Figure 2:**
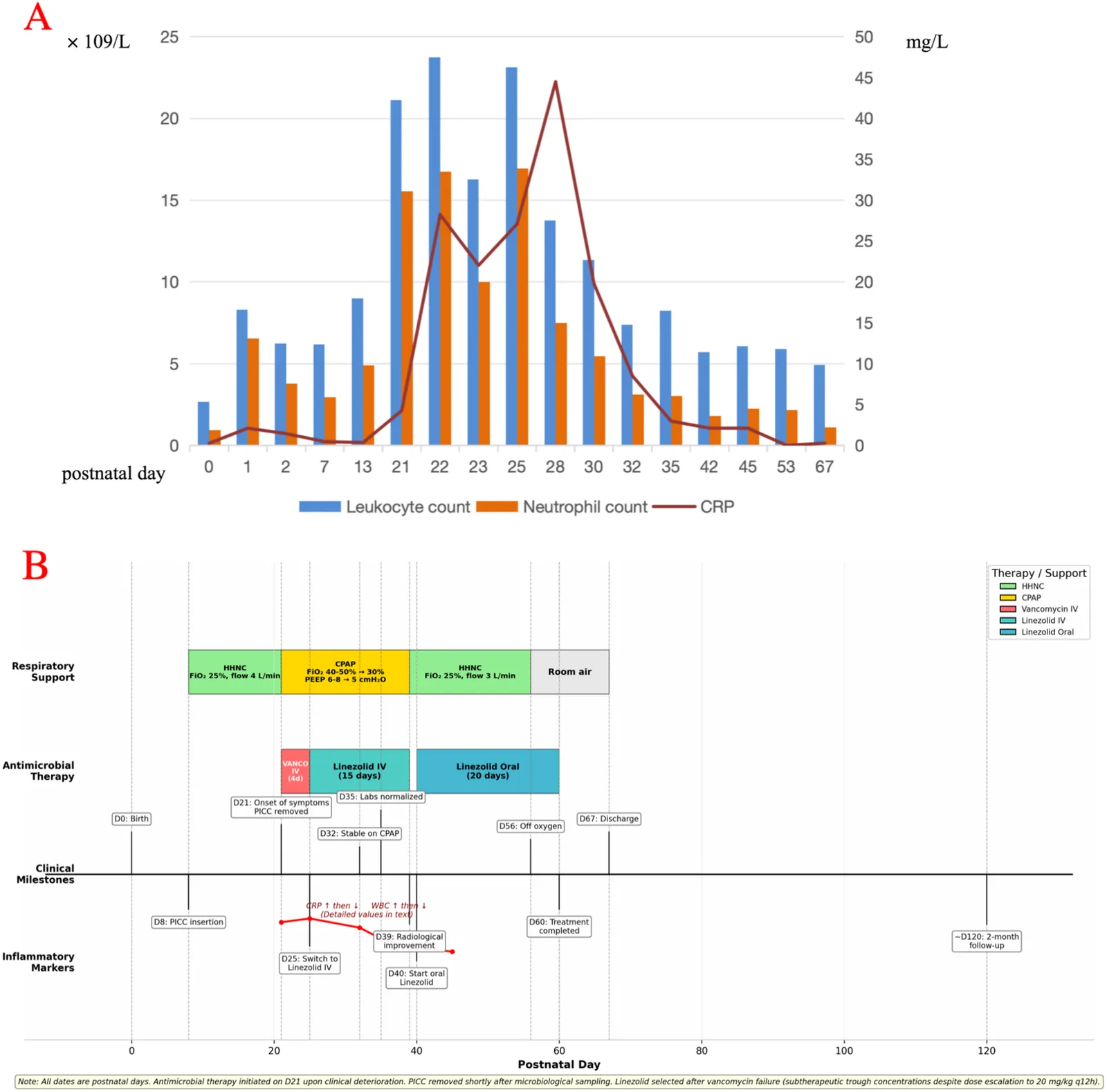
The clinical time line. **(A)** Changes in Leucocyte, neutrophils, and CRP. **(B)** Clinical Timeline of a Neonate with Presumed PICC-Related Septic Pulmonary EmbolismTotal Antimicrobial Therapy: 39 days (Vancomycin 4d + Linezolid IV 15d + Linezolid Oral 20d).

## 3 Discussion

Septic pulmonary embolism is a critical condition caused by infected thromboemboli obstructing the pulmonary arteries, leading to suppurative infection and infarction. Its clinical presentation is often nonspecific and can mimic pneumonia, making diagnosis challenging (9). SPE is rare in children and even more uncommon in neonates; due to its subtle symptoms, it is frequently missed or misdiagnosed, and its true incidence is likely underestimated. The diagnosis of SPE was established based on the criteria proposed by Cook et al. (10), and our case met all four criteria: (1) CT findings of bilateral peripheral thick-walled cavitary nodules; (2) PICC tip culture positive for MSSA, with NGS confirming the same organism in PICC and peripheral blood; (3) negative peripheral blood cultures, no vegetations on echocardiography, and negative testing for other pathogens (including tuberculosis, viruses, and fungi); (4) complete clinical and radiographic recovery after 35 days (5 weeks) of targeted linezolid therapy, preceded by 4 days of empirical vancomycin (total 39 days).

The characteristic imaging findings of large, thick-walled cavities may be attributed to the unique anatomical features of neonatal pulmonary vasculature. The small caliber of peripheral pulmonary arteries predisposes to the entrapment of septic emboli, leading to localized infarction and abscess formation (11, 12). This case emphasized that pulmonary imaging enables early detection of this rare complication in neonates with a history of central venous catheterization who present with unexplained respiratory deterioration (13). From an infectious microbiology perspective, the fact that both catheter-related NGS and cultures were positive revealed that PICC biofilms might serve as an occult infectious reservoir, with dislodged septic emboli causing focal pulmonary lesions but not leading to systemic bloodstream infection.

In this case, NGS was used as an adjunctive diagnostic tool and demonstrated its unique value (14). By directly detecting pathogen nucleic acids, NGS provided results within 24 h, substantially faster than conventional culture methods, facilitating early pathogen identification and targeted therapy. Particularly in this premature, low birth weight infant, early and precise diagnosis was critical for guiding effective antimicrobial treatment. Although *β*-lactams are first line agents for confirmed MSSA infections, vancomycin was chosen empirically at symptom onset because the causative pathogen was unknown and the infant had multiple risk factors (prematurity, low birth weight, central venous catheterization), considering the local infection epidemiology. Despite rapid microbiological confirmation of MSSA, the infant showed no clinical improvement, and vancomycin trough concentrations remained subtherapeutic despite dose adjustments, reflecting interindividual pharmacokinetic variability in neonates, particularly extremely low birth weight infants. This finding highlights the need for close therapeutic drug monitoring when vancomycin is used in this population. Subsequent switch to linezolid resulted in clinical and radiographic improvement. Furthermore, the negative lumbar puncture findings excluded meningitis at the time of presentation, supporting that the pulmonary lesions were the primary septic focus without concomitant central nervous system infection. Subsequent switch to linezolid resulted in clinical and radiographic improvement. We do not claim failure of *β*-lactam therapy, as these agents were not administered. Instead, the decision to use the prolonged IV-to-oral linezolid regimen was based on our institutional experience and established antimicrobial stewardship protocol, which favors linezolid for severe catheter-related *MSSA* infections in extremely preterm infants, particularly when prolonged parenteral access is impractical. This individualized approach, reached after multidisciplinary assessment, does not imply that linezolid should replace *β*-lactams as standard first-line therapy for MSSA. This underscores the importance of timely diagnosis, early recognition of risk factors, and adequate antimicrobial therapy in the management this situation.

Several differential diagnoses were considered and excluded. Severe necrotizing pneumonia (15–17) typically presents with consolidative, lobar, or segmental distribution, whereas the bilateral, multifocal, randomly distributed peripheral thick-walled cavities in our case suggest hematogenous dissemination (18). Although direct embolic evidence was lacking, the presence of a probable intravascular source (positive PICC culture), bilateral randomly distributed pulmonary lesions, and exclusion of other embolic sources support SPE as the most plausible mechanism (19, 20). Fungal infection and other causes of neonatal cavitary lung disease were ruled out by negative screening tests.

This report has several limitations. The diagnosis of SPE was clinicoradiological without histopathological or angiographic confirmation. Additionally, the entire catheterized venous pathway was not systematically assessed by Doppler ultrasound, which might have provided further evidence regarding the source of emboli. Negative conventional blood cultures raise the possibility that NGS detected bacterial DNAemia rather than true bacteremia. A positive PICC-tip culture may reflect colonization rather than true infection, as semiquantitative culture or differential time-to-positivity was not performed. Finally, NGS cannot establish clonal identity; without molecular strain-typing, we cannot prove that the PICC and pulmonary isolates originated from the same strain. These factors collectively temper the certainty of our conclusions.

## 4 Conclusion

This study reports a rare case of bilateral pulmonary abscesses secondary to presumed PICC-related septic pulmonary embolism in an extremely low birth weight infant. Microbiological evidence suggested that the PICC surface biofilm was the most probable source of septic emboli, leading to localized pulmonary infection without concurrent systemic bacteremia. This case highlights that SPE should be considered in the differential diagnosis for neonates with indwelling PICC who present with unexplained respiratory deterioration. Contrast-enhanced chest CT, combined with comprehensive pathogen detection (including NGS), facilitates early diagnosis and targeted therapy. Furthermore, these findings underscore the need for rigorous PICC maintenance and infection control strategies in the NICU to minimize biofilm formation and mitigate the risk of such severe complications.

## Data Availability

The original contributions presented in the study are included in the article, further inquiries can be directed to the corresponding author.
